# A Case of Asymptomatic Pulmonary Nocardia cyriacigeorgica Infection With Mild Diabetes Mellitus

**DOI:** 10.7759/cureus.24023

**Published:** 2022-04-11

**Authors:** Yumi Tsuchiya, Morio Nakamura, Tomoyo Oguri, Daisuke Taniyama, Shinji Sasada

**Affiliations:** 1 Department of Pulmonary Medicine, Tokyo Saiseikai Central Hospital, Tokyo, JPN; 2 Department of Pulmonary Medicine, National Hospital Organization Kanagawa Hospital, Kanagawa, JPN; 3 Department of Clinical Oncology, St. Marianna University School of Medicine, Kanagawa, JPN; 4 Department of Infectious Diseases, Showa General Hospital, Tokyo, JPN; 5 Department of Pulmonary Medicine, The Fraternity Memorial Hospital, Tokyo, JPN

**Keywords:** abnormal chest shadows, diabetes mellitus, bronchiectasis, nocardia cyriacigeorgica, pulmonary nocardiosis

## Abstract

Nocardiosis is a relatively rare opportunistic infection, ranging from localized to systemic diseases, commonly occurring in immunocompromised patients with high mortality rates. We present a case of a 61-year-old man who received medical treatment for type 2 diabetes mellitus and underwent a physical examination that showed abnormal chest shadows on radiography. Chest computed tomography revealed bronchiectasis and infiltration in the left lower lobe. *Nocardia spp.* was detected in the bronchial washes, and he was started on sulfamethoxazole-trimethoprim under the diagnosis of pulmonary nocardiosis. 16S ribosomal RNA gene sequencing analysis identified the species as *Nocardia cyriacigeorgica.* His pulmonary lesions successfully improved after treatment for six months. Pulmonary nocardiosis often presents with symptoms such as hemoptysis and blood-tinged sputum, and bronchiectasis has been identified as an underlying condition. Even in hosts without underlying immunocompromising conditions, *Nocardia spp.* can be a causative microorganism of pulmonary infections, and it should be considered in the differential diagnoses.

## Introduction

*Nocardia spp.* are aerobic gram-positive filamentous rods that are widely distributed in the environment, including various soil, water, and organic matter habitats. The inhalation of airborne droplets, which most commonly causes pulmonary nocardiosis, is the most common route of infection. *Nocardia* infection is opportunistic in cases of administration of immunosuppressive drugs, such as corticosteroids after organ transplantation, and in hosts with reduced cellular immunity, such as acquired immunodeficiency syndrome, cancer, lupus, and diabetes [1]. Moreover, chronic respiratory diseases such as chronic obstructive pulmonary disease and bronchiectasis are considered the underlying conditions of pulmonary nocardiosis [2]. Mainly the lungs and the skin are infected; however, the infection may disseminate to the central nervous system. The central lesions are present in approximately 40% of disseminated cases and are associated with a poor prognosis, with a mortality rate of 30-40% [3]. We report a case of asymptomatic pulmonary nocardiosis caused by *Nocardia cyriacigeorgica* with underlying bronchiectasis and mild diabetes mellitus.

## Case presentation

A 61-year-old man undergoing treatment for type 2 diabetes mellitus was diagnosed with an abnormal chest shadow during physical examination. He has smoked 27 pack-years without a history of pet keeping or dust exposure. Chest computed tomography (CT) showed bronchiectasis and infiltration in the left lower lobe, which worsened after four months. The patient was then admitted for bronchoscopy.

**Figure 1:**
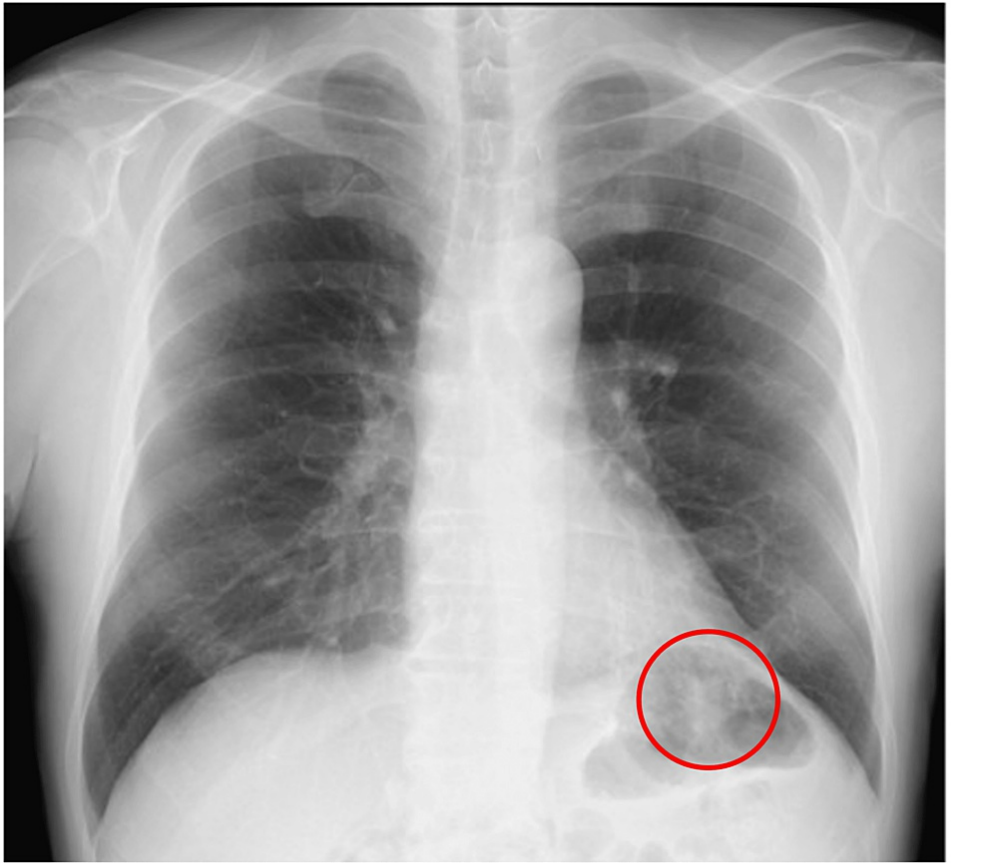
Chest radiograph on admission for a bronchoscopy An infiltrative shadow in the left lower lung field was demonstrated (red circle).

**Figure 2:**
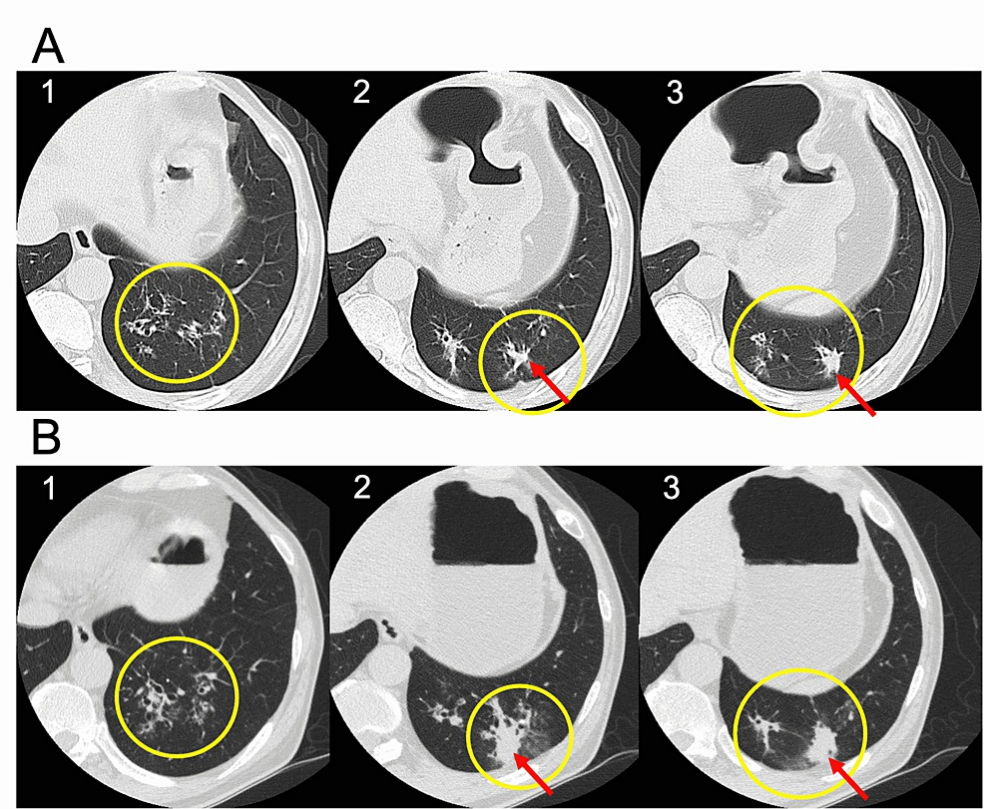
Chest computed tomography on the first examination (A) and on the admission for a bronchoscopy (B) In four months from the first examination (A) to the admission for a bronchoscopy (B), infiltrative and ground glass shadows around bronchiectasis (1) and nodules (2, 3) worsened (yellow circles), and nodular lesions increased in size (2, 3) (red arrows) in the left lower lung lobe.

Upon examination, the patient was afebrile with stable vital signs. On admission, laboratory findings showed no elevation of white blood cells or C-reactive protein levels. His blood glucose level was elevated to 139 mg/dL, and his hemoglobin A1c level was 7.2%. Immunoglobulin levels were average, and human immunodeficiency virus antibody and human T-cell lymphoma virus-1 antibody were negative. The interferon-γ release assay was negative. Sputum bacteriological examination revealed only normal respiratory flora, and both smear and culture were negative for acid-fast bacilli. Chest radiograph showed an infiltrative shadow in the left lower lung field (Figure 1), and chest CT revealed the enlargement of the infiltrative and nodular shadows with ground-glass opacity and bronchiectasis in the left lower lung lobe (Figures 2A, 2B). Transbronchial biopsy and bronchial wash with bronchoscopy were performed on the left B10c because peripheral radial ultrasound bronchoscopy did not reveal any blood vessels near the puncture site. The patient was discharged the next day without any major early complications but was readmitted for hemoptysis seven days after bronchoscopy.

Gram staining of the bronchial washes revealed negative results. However, the culture showed the growth of *Nocardia spp*. Histopathological findings of the biopsy specimens showed fibrosis of the interstitium under the bronchial mucosa and inflammatory cell infiltration, with non-neoplastic lesions. The species was identified as *N. cyriacigeorgica* by 16S ribosomal RNA (rRNA) gene sequencing analysis. Antimicrobial susceptibilities were as follows: susceptible: trimethoprim/sulfamethoxazole, amikacin, ceftriaxone, imipenem, linezolid, tobramycin, cefotaxime, cefepime, and gentamicin; intermediate: minocycline and doxycycline; resistant: amoxicillin/clavulanate, ciprofloxacin, and clarithromycin.

No lesions in other organs, including the central nervous system, were detected. Under the diagnosis of pulmonary *N. cyriacigeorgica *infection, a sulfamethoxazole-trimethoprim combination of 12.0 g/day (15 mg/kg trimethoprim) was started. After starting the medication, anorexia and malaise appeared markedly, making it challenging to continue the oral therapy.

The dosage of the medication was reduced to 6 g/day. Subsequently, the symptoms alleviated, and the patient was able to take medication without any recurrence of hemoptysis. After six months of treatment, a chest CT scan showed significant shrinkage, improved lesions, and no new lesions. Treatment was terminated, and no recurrence of the disease was observed.

## Discussion

Pulmonary nocardiosis is caused by the inhalation or aspiration of *Nocardia spp*. In Japan, the *N. asteroides complex* (*N. asteroides sensu stricto, N. farcinica, and N. nova*) accounts for approximately 90% of the causative microorganisms, and *N. cyriacigeorgica,* which was detected in this case, is relatively rare. The genus *Nocardia* was differentiated into five species, *N. asteroides, N. brasiliensis, N. farcinica, N. nova, and N. cyriacigeorgica*, based on their susceptibility to antimicrobial agents (imipenem, tobramycin, kanamycin, and 5-fluorouracil). However, more species have recently been discovered, and it has become difficult to identify species based on drug susceptibility alone [4].

In recent years, identification of bacterial species using 16S rRNA gene sequencing analysis has been conducted at limited facilities, and changes in the distribution and trends of causative microorganisms have been observed. According to a study on pulmonary nocardiosis by Ishiguro et al., in 16 cases, *N. asteroides* was the most common species identified using previous identification methods [5]. Of the seven cases for which 16S rRNA gene sequencing analysis was performed, two cases initially identified as *N. asteroides* and one identified as *Nocardia spp.* were re-identified as *N. cyriacigeorgica*. Using molecular methods, a significant proportion of the *N. asteroides* complex has been identified to be *N. cyriacigeorgica* [6]. In a review of pulmonary nocardiosis in Taiwan by Chen et al., *N. cyriacigeorgica* was the most common causative organism [7]. 16S rRNA gene sequencing analysis was used to identify the organism responsible for pulmonary nocardiosis, and it was suggested that *N. cyriacigeorgica* might no longer be a rare organism. An increased number of *N. cyriacigeorgica* infections has been reported in patients with diabetes and with the administration of immunosuppressive drugs [8]. However, in an epidemiological survey in the United States, *N. nova* was the most common species responsible for nocardiosis. This indicates that the frequency and distribution patterns of each species may differ among countries and regions [9].

Moreover, it has been reported that diseases caused by *N. cyriacigeorgica* are more severe than those caused by other species [7]. In this case, the lesion was incidentally detected during the physical examination. It later caused hemoptysis; however, the initial treatment was successful, and the patient had a relatively good clinical course.

Pulmonary nocardiosis is commonly observed as an opportunistic infection in immunosuppressed patients. In a study of 43 cases of pulmonary nocardiosis in western European countries, 83.7% were reported to have depressed cellular immunity [10]. In contrast, approximately 36% of *Nocardia* infections occur in immunocompetent patients [11]. Chronic respiratory diseases, such as chronic obstructive pulmonary disease, bronchiectasis, and cystic fibrosis, are the primary predisposing conditions for pulmonary nocardiosis, and a decrease in the self-cleaning function of the local airway in these diseases is involved in the development of infection [2]. However, *Nocardia spp*. maybe temporarily coexisted in the respiratory tract of patients with chronic respiratory diseases, and a comprehensive assessment, including the clinical course, is necessary to determine whether it is the causative organism or not [12,13].

In a review of 55 cases of pulmonary nocardiosis [14], complications were observed in 58.2% of patients. Septicemia and respiratory failure were most common (46.8%). Dissemination also commonly occurred in 31.2% of patients. This review also reported a mortality rate of 34.5% for this disease. The common causes of mortality included septicemia or respiratory failure (57.8%) and pulmonary embolism (10.5%). In patients with dissemination, mortality was 100%.

The symptoms of pulmonary nocardiosis are nonspecific, and the most common symptoms are cough, shortness of breath, and fever; however, hemosputum or hemoptysis may also occur. This disease often remains undiagnosed because of its relatively nonspecific clinical symptoms, radiological presentations ranging from nodules, cavitation, and ground-glass opacities, and the low diagnostic yield of clinical specimens obtained using noninvasive procedures [15,16]. A delay in diagnosis may be responsible for treatment failure and a high mortality rate.

The most common therapy for nocardiosis is trimethoprim-sulfamethoxazole. Based on drug susceptibility, other antibiotics including amikacin, ceftriaxone, levofloxacin, linezolid, imipenem, and amoxicillin-clavulanic acid may be considered. The standard treatment duration is 6-12 months; however, it may be prolonged in immunocompromised patients [17].

Clinicians may underestimate the burden of pulmonary nocardiosis, and it is essential to consider nocardiosis as a possible cause of new abnormal chest shadows in patients with chronic respiratory disease, even in the absence of obvious immunosuppression.

## Conclusions

We report a case of pulmonary nocardiosis caused by *N. cyriacigeorgica*, which was detected on physical examination. Based on the background of the underlying diabetes mellitus and bronchiectasis, this case was considered a high-risk case. *Nocardia spp*. should be regarded as potential causative microorganisms, even in hosts without underlying severe diseases. As pulmonary nocardiosis is associated with a high mortality rate, early diagnosis and treatment may lead to a successful recovery of the affected patients.
